# Impact of osmotic stress on the growth and root architecture of introgression lines derived from a wild ancestor of rice and a modern cultivar

**DOI:** 10.1002/pei3.10026

**Published:** 2020-08-08

**Authors:** Lin Chen, Tomasz Czechowski, Ian A. Graham, Sue E. Hartley

**Affiliations:** ^1^ Department of Biology University of York York UK; ^2^ Present address: Department of Animal and Plant Sciences University of Sheffield Sheffield S10 2TN UK

**Keywords:** abscisic acid, drought resilience, introgression lines, osmotic stress, root architecture, shoot growth, wild ancestor of rice

## Abstract

Many modern rice varieties have been intensively selected for high‐yielding performance under irrigated conditions, reducing their genetic diversity and potentially increasing their susceptibility to abiotic stresses such as drought. In this study, we tested benefits for stress tolerance of introducing DNA segments from wild ancestor *Oryza rufipogon* to the modern cultivar *O. sativa* cv *Curinga* (*CUR*) by applying a gradient of osmotic stress to both parents and seven introgressed lines. Shoot growth of *O. rufipogon* had a high tolerance to osmotic stress, and the number of total root tips increased under mild osmotic stress. One introgression line showed greater shoot growth, root growth, and higher number of total root tips than the parent line *CUR* under osmotic stress. Abscisic acid (ABA) is a key hormone mediating plant responses to abiotic stresses. Both root and shoot growth of *O. rufipogon* were much more sensitive to ABA than *CUR*. Introgression lines varied in the extent to which the sensitivity of their growth responses to ABA and some lines correlated with their sensitivity to osmotic stress. Our results suggest that rice responses to ABA and osmotic stress are genotype dependent, and growth responses of rice to ABA are not a consistent indicator of resilience to abiotic stress in introgression lines.

## 1 INTRODUCTION

Asian rice (*Oryza sativa* L.) is the major staple crop for 3 billion people around the world (Lampe, 1995). To meet the increasing food demand from the growing world population, the UN Food and Agriculture Organisation estimated that rice production needs to increase 50% by 2030 (FAO, 2009). However, achieving this is challenging given the pressures from climate change and the unsustainable use of resources in many intensive agricultural systems. For example, drought is one of the most important global stresses for crops, resulting in up to 5–6% loss of global rice production between 1964 and 2007, while recent droughts have produced even greater losses (Lesk et al., 2016). In some regions rice cultivation faces additional challenges when rain is the only water source, as is the case for 56% of total area of rice cultivation in India (Singh et al., 2017) and more than 80% of that in Thailand (FAO, 2000). In these rain‐fed regions, the growth of rice is solely dependent on monsoon rain, which is becoming more erratic in some regions (Singh et al., 2017). Crop varieties which can maintain their yield in the face of these changing and unpredictable climatic conditions offer great potential to address food security challenges (Macholdt and Honermeier, 2016). However, intensive agricultural breeding has produced modern rice varieties which have high yield potential, but reduced genetic diversity, often associated with greater susceptibility to biotic and abiotic stresses (Tanksley and McCouch, 1997). For example, a widely grown modern variety IR64, bred to gain high yield in irrigated paddy areas, is very susceptible to drought as it has a shallow root system (Clark et al., 2011). Landraces and crop wild relatives which can grow in more variable environmental conditions than modern cultivars can provide natural repositories of genetic diversity, thus they can be used to improve the resilience of crops to the changing climate (McCouch et al., 2013). For example, Uga et al. (2013) introduced a quantitative trait locus (QTLs) (DRO1) from landrace Kinandang Patong to IR64 to produce a variety with increased deep rooting and the ability to produce higher yield under drought conditions.

A vigorous root system with early and rapid root extension and proliferation allows plants to access more water from the soil (Dodd et al., 2010; Richards, 2008). In maize and rice, root elongation can be stimulated by early mild soil drying, and is inhibited when the soil drying progresses to severe (Kano et al., 2011; Sharp and David, 1989; Watts et al., 1981). Therefore, rice cultivars which can maintain root growth at the early stage of drought are better able to access water in the deeper soil and more likely remain productive through the drought period, particularly in the earlier stages when young plants face an early spring drought (Fukai and Cooper, 1995). Wild species *Oryza rufipogon*, the ancestor of cultivated rice, was widely distributed across Asia from several thousand years ago (Gross and Zhao, 2014). It has very different morphological and physiological traits from modern domesticated rice cultivars and is adapted to a wider range of environments. Therefore, *O. rufipogon* could be a source of root growth‐related genes to increase rice resilience (Atwell et al., 2014; Vaughan et al., 2008). For example, Hu et al. (2007) reported that Dongxiang wild rice (*O. rufipogon* Griff.) had more roots and survived better than cultivated rice after exposure to drought at the seedling stage.

Responses of root growth to stresses, including drought, are mediated by plant hormones such as abscisic acid (ABA) (De Smet et al., 2006; Verslues and Zhu, 2005; Wilkinson and Davies, 2002). Water stress stimulates the accumulation of ABA in plant roots (Ding et al., 2016; Puértolas et al., 2015; Xu, Jia et al. 2013; Zhang and Davies, 1989; Zhang and Davies 1989), and the concentration of ABA in plant roots could be an indicator for the local change of soil water potential (Zhang and Davies, 1989). Interestingly, ABA has dual effects on root growth: relatively low concentrations of ABA can stimulate primary root growth, while relatively high concentrations inhibit primary root growth (Li et al., 2017; Watts et al., 1981; Xu et al., 2013). Xu et al. (2013) suggested that ABA regulates the stimulation response of root growth to mild osmotic stresses. Therefore, differences in the sensitivity of modern cultivars and wild ancestors to ABA may underpin differences in their resilience to abiotic stresses such as drought.

Chromosome segment substitution lines (CSSLs) are commonly used to map quantitative trait loci (QTLs), identify segments associated with specific QTLs and study gene interactions in rice (Kubo et al., 2002). *Oryza rufipogon* has previously been used as a donor to construct introgression lines in a cultivated rice background (Tian et al., 2006). An introgression line bred from *O. rufipogon* genotype Dongxiang and *O. sativa* showed an increased survival rate of plants under drought conditions (Zhang et al., 2014). However, some parameters from *O. rufipogon* and its introgression lines such as root architecture or depth of roots, which are crucial for water uptake (Ahmed et al., 2016; Kato and Okami, 2011), have not so far been studied. Furthermore, it is also unknown whether *O. rufipogon* and its introgression lines have different responses to drought in terms of shoot and root growth, or it is clear whether there is any correlation between the growth response to drought and to ABA in *O. rufipogon* and its introgression lines. A good understanding of the effect of *O. rufipogon* introgression on shoot biomass accumulation, root architecture, and ABA responsiveness of the root system under osmotic stress would be beneficial to breeders aiming to enhance the crop performance under abiotic stresses by helping to identify quantitative trait loci for desirable root traits.

Our study aimed to support the development of drought‐tolerant lines suitable for rain‐fed agriculture in India, where increasingly erratic rainfall has the capacity to severely curtail yields (Singh et al., 2017) and where hard pan caused by soil drying can greatly limit the penetration of roots in deep soil layers (Fukai and Cooper, 1995). To maximize the possibility of obtaining new traits for drought tolerance to support the development of new lines for Indian farmers in upland rain‐fed areas, we used CSSLs produced from the tropical japonica upland cultivar *O. sativa* cv *Curinga* (*CUR*) as the recipient and *O. rufipogon* as the donor (Arbelaez et al., 2015). Marker‐assisted selection (MAS) had been used to demonstrate that each introgression line contained a few well‐defined chromosomal segments from *O. rufipogon* in *CUR* background. We focused on the plant growth response to osmotic stress in the vegetative stage, having compared drought tolerance in *O. rufipogon* and the modern cultivar *CUR* in terms of shoot biomass accumulation. We went on to test for beneficial traits of deep rooting, higher number of total root tips and improved shoot biomass accumulation under osmotic stress in the introgression lines. The difference in root architecture in response to osmotic stress between *O. rufipogon* and *CUR* was also analyzed and compared with the difference in their response to ABA.

## 2 MATERIALS AND METHODS

### 2.1 Plant materials and growth conditions

*Curinga* (*O. Sativa ssp. tropical japonica*) was developed by the Empresa Brasileira de Pesquisa Agropecuaria (EMBRAPA, Goiania Brazil) for commercial purposes (Arbelaez et al., 2015; Morais et al., 2005). The *O. rufipogon* line used here was *O. rufipogon* Griff. Acc. IRGC 105491 (RUF) (International Rice Research Institute, IRRI; http://www.irgcis.irri.org:81/grc/IRGCISHome.html) from Kelantan, Malaysia; *O. rufipogon* widely grows in many different parts of the world. The CSSLs derived from *CUR* and *O. rufipogon* were genotyped by genotyping by sequencing (GBS) and SSRs (Arbelaez et al., 2015). The 48 introgression lines which collectively contain 97.6% of the donor genome with an average of 89.9% recurrent parent genome per line were kindly provided by Prof Susan McCouch of Cornell University, US. Rice seeds were sown into the terragreen 0.5 cm deep. The terragreen was soaked in water and kept well watered during the germination. Seeds were germinated in the glasshouse at conditions: temperature of 28°C for the day and 22°C for the night. High pressure sodium lamps were used to give light intensity of 170 µmol m^2^ s^−1^ for 12 hours photoperiod. Ten days after sowing, uniform germinated rice seedlings from each line were carefully transferred to a hydroponic system containing 9L nutrient solution. Rice seedlings were cultured with Magnavaca nutrient medium (Magnavaca et al., 1987) for 4 days. The pH of solution was adjusted to 5.5 by using KOH or HCl.

### 2.2 ABA and PEG treatments

ABA (A1296, Sigma‐Aldrich) or polyethylene glycol 6000 (PEG 6000‐26603, VWR Ltd, Lutterworth LE17 4XN, UK) treatments were applied to 14‐day‐old seedlings with renewed nutrient solution. The pH was again adjusted to 5.5 after the treatments. ABA stock solutions were made in 10 mM (±ABA) with 0.03 M KOH.

### 2.3 Physiological measurements

PEG 6000 solutions at concentrations of 5%, 10%, and 15% and ABA solutions at concentrations of 0.001, 0.1, and 5 µM were applied to study their effects on root growth, including the longest root length, the total root length, and the number of total root tips. According to previous calculations (Michel and Kaufmann, 1973), 5%, 10%, and 15% PEG 6000 are equivalent to the osmotic pressure at −0.05, −0.15, and −0.3 MPa, respectively. ABA concentrations at rice (*Oryza sativa* L. Nipponbare) root tips were around 110 ng g^−1^ FW, approximately equivalent to a tissue concentration of 0.5 µM (Xu et al., 2013). Seedlings at 14 days old were transferred to growth medium with or without PEG or ABA for 3 days. Roots of each seedling were scanned using an Epson V700 scanner, and total root length and the number of total root tips were measured by using a root analysis instrument (WinRHIZO; Regent Instruments Inc., Quebec, ON, Canada). Shoot and root biomass were measured after oven drying.

### 2.4 Statistical analysis

The software GenStat 18.2.0 was used to perform one‐way ANOVA and multiple pairwise comparisons with Tukey’s post hoc test at the *P* < .05 level for the analysis of the root morphology data. The software SigmaPlot 13.0 was used to analyze all other data by one‐way ANOVA and multiple pairwise comparisons with Tukey’s post hoc test at the *P* < .05 level, and significant correlation at the *P* < .05 level.

## 3 RESULTS

### 3.1 Growth responses of shoot and root to PEG‐induced osmotic stress

**FIGURE 1:**
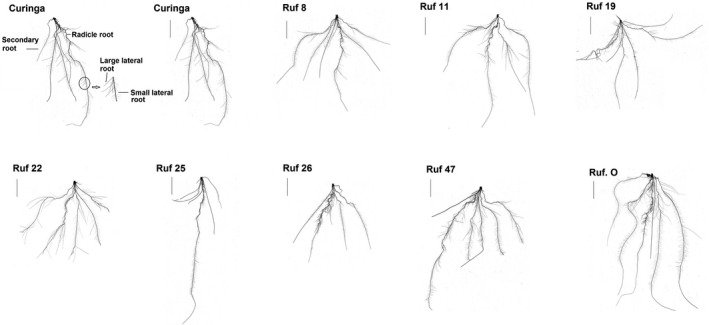
Morphology of the embryonic root system of seedling *CUR*, 1 week after germination, and the morphology of the rice genotypes used in the experiments: *CUR*, *O. rufipogon,* and CSSLs *CUR/RUF* 8, 11, 19, 22, 25, 26, and 47. The scale bar indicates 10 cm

**FIGURE 2:**
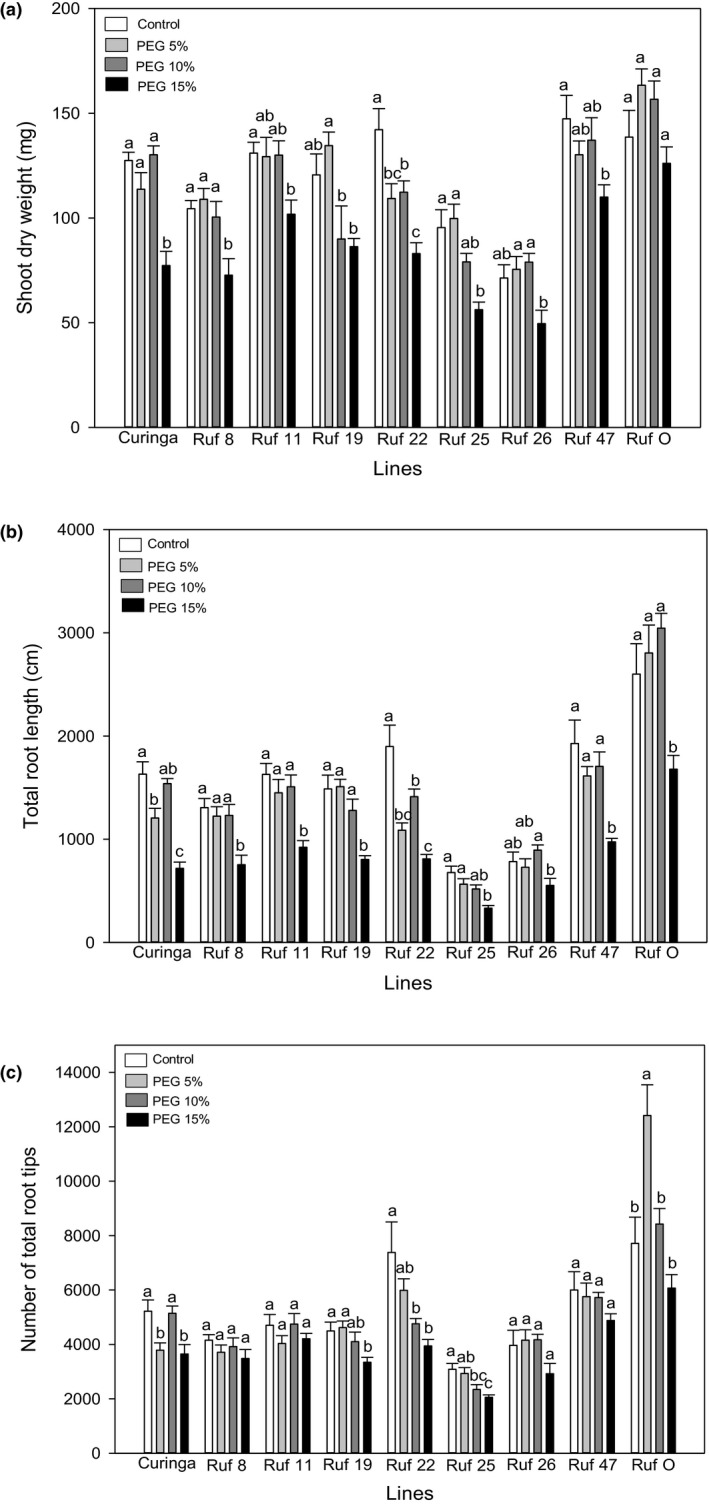
Responses of (a) the shoot dry weight, (b) the total root length, and (c) the number of total root tips to control, 5%, 10%, and 15% PEG treatments in *CUR*, *O. rufipogon,* and seven CSSLs. The values are means, and the vertical bars represent standard errors. Data analyzed using one‐way ANOVA and different letters indicate significant differences between three PEG treatments at *p* < .05. n = 5 or 6

Seven of the forty‐eight fixed *CUR/RUF* introgression lines (*CUR/RUF* 8, 11, 19, 22, 25, 26, and 47) were selected for their contrasting root phenotypes (Figure 1). For example: *CUR/RUF* 25 had an unusual root phenotype with short secondary and lateral roots compared to other lines; all roots in *CUR/RUF* 26 were relatively short; *CUR/RUF* 22 and 47 had large root systems in comparison to other CSSLs; *O. rufipogon* had the longest radicle and secondary roots across all lines. These differences were found to be statistically significant when the lines were grown under control conditions (Figure 2b,c, and Figure S1a). The total root length of *CUR/RUF* 25 and 26 was significantly less than *CUR*, *CUR/*RUF 11, 22, and 47 and *O. rufipogon*, whereas the root length of *CUR/RUF* 22 and 47 did not differ significantly from *O. rufipogon* (One‐way ANOVA; Tukey's test *P* < .05). Similarly, *CUR/*RUF 25 had fewer root tips than all other lines, significantly differed from *CUR/*RUF 22 and 47 and *O. rufipogon* (One‐way ANOVA; Tukey’s test *P* < .05). Except *CUR/RUF* 22, all other lines had a significantly larger longest root than *CUR/RUF* 26; there was no significant difference between the longest root of *CUR/RUF* 25 and 47, and *O. rufipogon* (One‐way ANOVA; Tukey’s test *P* < .05).

*O. rufipogon* showed a significantly higher shoot biomass compared to all other lines apart from *CUR/RUF* 19 under the 5% PEG treatment, indicating its high tolerance to osmotic stress (Table S1). *CUR/RUF* 19 and 26 had high tolerance to the imposed osmotic stress, similar to that of *O. rufipogon*. The treatment of 15% PEG concentration had no significant impact on the shoot dry weight of these three lines but it inhibited the shoot dry weight of all other lines (Figure 2a). *CUR/RUF* 47 showed a better performance of shoot dry weight under 15% PEG treatment than *CUR* (*t* test, *P* < .05). The 15% PEG treatment caused a 39% decrease in shoot dry weight in the *CUR* parent, but only a 25% reduction in *CUR/RUF* 47 (Figure 2a). The average of shoot dry weight of *CUR/RUF* 47 was 43% higher than that of *CUR* after the 15% PEG treatment. *CUR/RUF* 22 was the most sensitive line to the PEG treatments: its shoot dry weight was significantly reduced (by 19%) under the 5% PEG treatment (Figure 2a), which had no significant impact on other lines (Table S2).

The highest PEG treatment at 15% significantly inhibited the total root length of all lines except *CUR/RUF* 26 (Figure 2b; Table S2). Only the 15% PEG treatment was a sufficient stress to significantly inhibit the total root length of *O. rufipogon* but even under these conditions its root length was similar to that of *CUR* grown in the absence of PEG (Figure 2b). *O. rufipogon* also showed a significantly higher total root length than all other lines in the 10% PEG treatment (Figure 2b; Table S1). Similar to the shoot response to PEG treatments, the total root length of *CUR/RUF* 47 was significantly higher (36%) than *CUR* under the 15% PEG treatment (*t* test, *P* < 0.05). PEG treatments had a pronounced inhibitory effect on the total root length of *CUR/RUF* 22, again matching the effects on its shoot biomass (Figure 2a); even the 5% PEG treatment significantly inhibited the growth of root length in this line, by around 43% (Figure 2b).

The impact of the PEG treatments on the number of total root tips of *O. rufipogon* (Figure 2c; Table S2) differed from that of *CUR* and all introgression lines. The number of total root tips of *O. rufipogon* was significantly stimulated by the 5% PEG treatment and not significantly inhibited by the 15% PEG treatment. The highest PEG treatment significantly inhibited the number of total root tips of *CUR*, but did not affect *CUR/RUF* 47; this line had significantly higher number of total root tips (30%) than *CUR* under the 15% PEG treatment (*t* test, *P* < 0.05). The number of total root tips of *CUR/RUF* 22 and 25 reduced more to PEG treatments than other lines: 10% PEG significantly inhibited their root tip numbers. Compared to the number of total root tips, the growth of the longest root was very sensitive to PEG treatments in most lines (Figure S1a). For example, the 15% PEG treatment inhibited the length of the longest root in all lines except *CUR/RUF* 19, 22, and 26. *CUR/RUF* 22 showed a tendency for increased length of the longest root under 5% and 10% PEG treatments, although this was not significant. Taken together, we found that *O. rufipogon* had significantly higher total root length and more root tips than these of *CUR* under the control, 10% and 15% PEG treatments (*t* test, *P* < .05). Although the 15% PEG treatment inhibited the total root length and the longest root length of *O. rufipogon*, they were still as high as those of *CUR* under the control treatment. *CUR/RUF* 26 was the only line in which the total root length, the number of total root tips, the growth of the longest root, and the shoot dry weight were not affected by PEG treatments (Figure 2a,b and Figure S1a).

**FIGURE 3:**
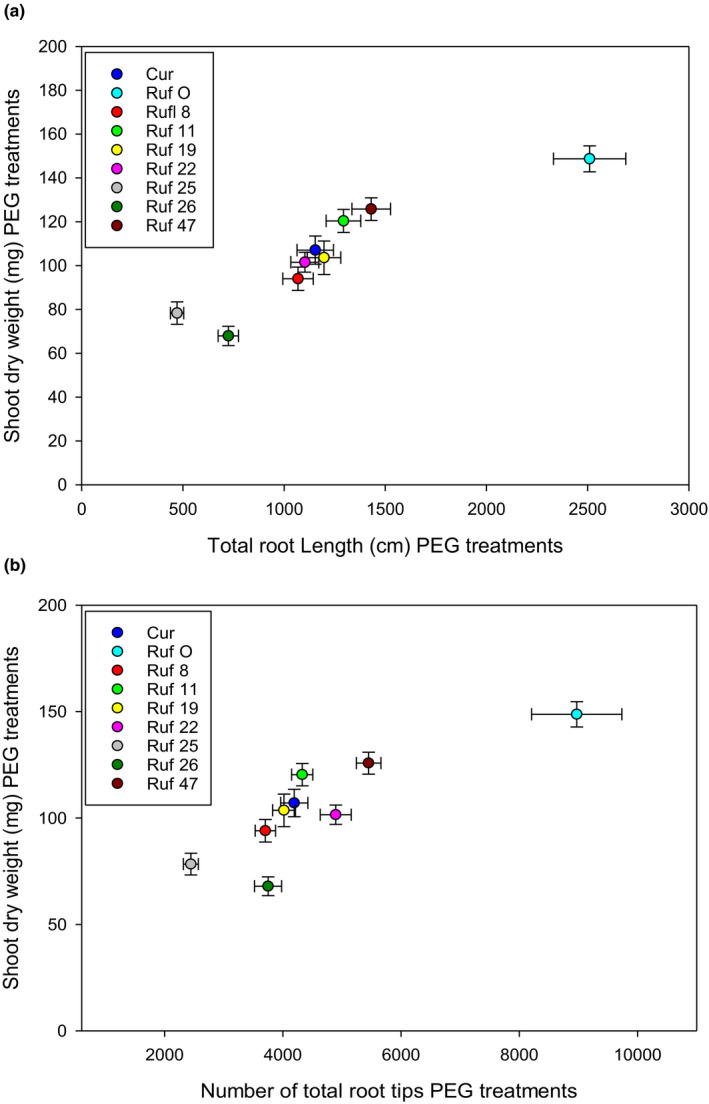
The correlation after PEG treatment between shoot dry weight and (a) the total root length and (b) the number of total root tips of all CSSLs. Note data are from combining data from across all three PEG treatments (5%, 10%, and 15%). The values are means, and the vertical bars represent standard errors

Our results suggest that the responses of shoot and root growth to PEG were related, particularly in lines *CUR/RUF* 22 and 47, and indeed we found positive correlations across lines between the shoot dry weight and either the total root length or the number of total root tips under all PEG treatments combined (*P* < .05) (Figure 3a and b).

### 3.2 Growth responses of shoot and root to ABA

**FIGURE 4:**
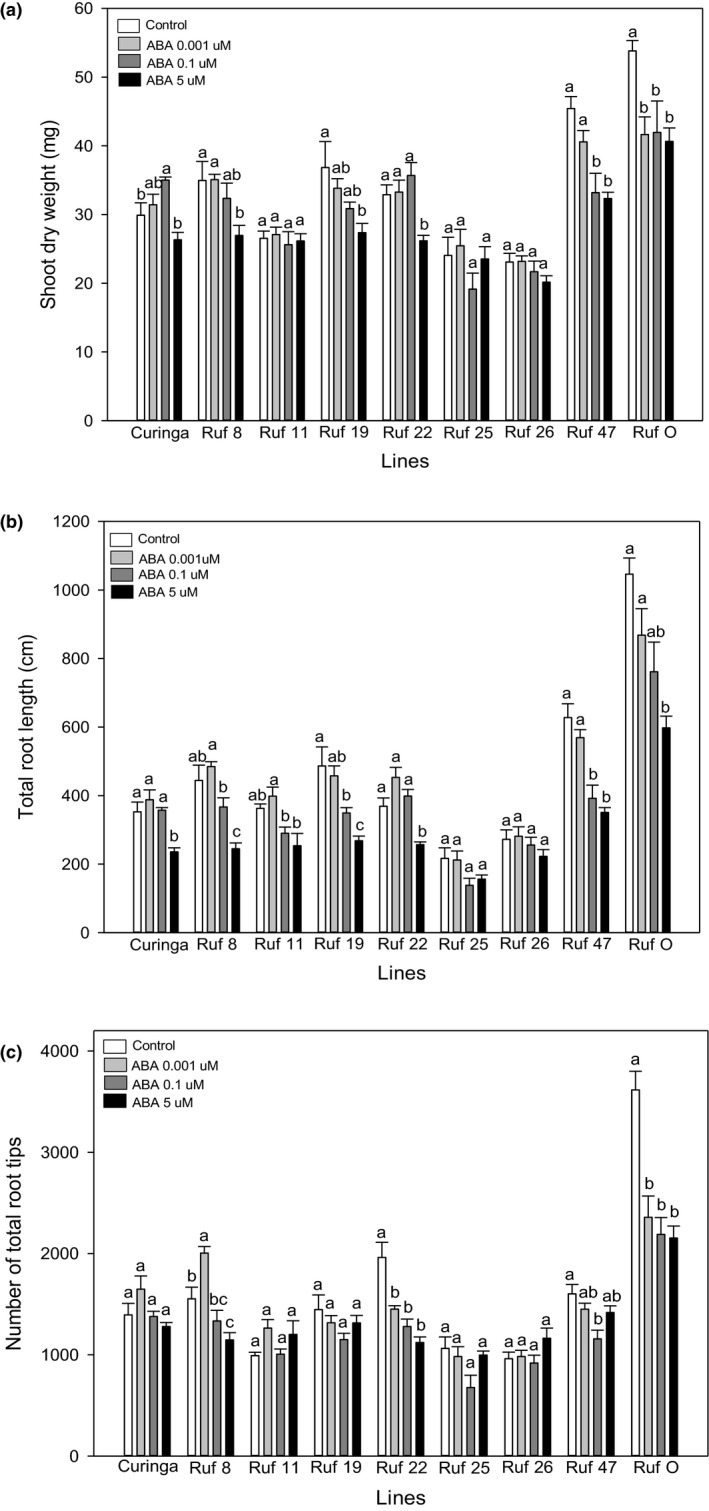
Responses of (a) the shoot dry weight, (b) the total root length, and (c) the number of total root tips to control, 0.001, 0.1, and 5 ABA treatments in *CUR*, *O. rufipogon,* and seven CSSLs. The values are means, and the vertical bars represent standard errors. Data analyzed using one‐way ANOVA and different letters indicate significant differences between three PEG treatments at *P* < .05. n = 5 or 6

*CUR* showed an interesting growth pattern of shoot dry weight in response to ABA treatments (Figure 4a). The relatively low concentration of ABA (0.1 µM) significantly stimulated the shoot growth of *CUR*, but a high concentration at 5 µM had no significant impact. However, its root growth did not reflect this pattern (Figure 4b,c). In contrast, *O. rufipogon* was very sensitive to ABA treatments. The ABA treatment at 0.001 µM had a significant inhibitory effect on its shoot dry weight, total root length and the number of total root tips (Figure 4a–c). ABA treatments had no effects (either stimulatory or inhibitory) on the accumulation of shoot dry weight of *CUR/RUF* 11, 25, and 26 (Figure 4a); their root growth (total length and the number of total root tips) showed similar patterns as their shoot response (Figure 4a–c and Table S2).

At least one concentration of ABA treatment had a significant inhibitory effect on the total root length of all lines apart from *CUR/RUF* 25 and 26 (Figure 4b; Table S2). Interestingly, *CUR/RUF* 26 was also entirely insensitive to PEG treatments as well (Figure 2a). The total root growth of *CUR/RUF* 19 and 47 was more sensitive to ABA treatments than that of other lines (Figure 4b): the ABA treatment at (0.1 µM) had a significant inhibitory effect on the total root growth of these two lines but not on that of others. For most lines, their number of total root tips did not respond to ABA treatments, with the exception of *O. rufipogon*, *CUR/RUF* 8 and 22 (Figure 4c; Table S2). The ABA treatment at 0.001 µM significantly inhibited the number of total root tips of *O. rufipogon* and *CUR/RUF* 22 by 35% and 26%, respectively. Interestingly, the same concentration of ABA significantly increased the number of total root tips of *CUR/RUF* 8, while the ABA treatment at 5 µM significantly inhibited it. The growth of the longest root of *CUR/RUF* 19 showed a high sensitivity to ABA treatments (Figure S1b): the ABA concentration at 0.001 µM significantly inhibited its longest root growth. The growth of the longest root of *CUR/RUF* 25 and 26 was insensitive to ABA treatments. This response pattern was also observed in their shoot growth, total root growth, and the number of total root tips under ABA treatments (Figure S1b; Figure 4a,b and Table S2).

### 3.3 Comparison of responses to osmotic stress and ABA

Although a stimulation effect on the shoot growth was observed in *CUR* under ABA treatments (Figure 4a), such effects did not occur in PEG treatments across all lines (Figure 2a). Shoot growth of all lines was more sensitive to PEG treatments than to ABA, as at least one PEG treatment had a significant inhibitory effect on the shoot growth of each line, including *CUR/RUF* 11, 25, and 26, which were insensitive to ABA treatments (Figures 2a and 4a). The shoot dry weight of *O. rufipogon* was insensitive to PEG treatment but very sensitive to ABA treatment, while *CUR/RUF* 22 showed a high sensitivity to PEG treatment, but a relatively low sensitivity to ABA treatments (Table S2). Both PEG and ABA treatments significantly inhibited the number of total root tips in *CUR/RUF* 22 (Figures 2c and 4c) possibly because it had a relatively small but highly branched root system compared to other lines (Figures 1, 2c and 4c). *CUR/RUF* 8 and 47 had similar growth pattern of shoot and total root length when subjected to ABA or PEG treatments (Figures 2a,b and 4a,b). The longest root growth of *CUR/RUF* 19 was very sensitive to ABA treatments compared with most other lines, while it was insensitive to PEG treatments (Figures S1a and b).

## 4 DISCUSSION

Our recipient parent *CUR* is one of the most drought‐tolerant modern cultivars (Arbelaez et al., 2015; Sakai et al., 2010), as evidenced in our experiment, where we found that only the higher level of osmotic stress (−0.3 MPa) significantly inhibited its shoot growth (Figure 2a). However, it was still outperformed by the wild ancestor *O. rufipogon*, which did not suffer any inhibitory effects under any of our osmotic stress treatments in terms of shoot growth. Furthermore, *O. rufipogon* also had the most vigorous root growth under both control treatment and osmotic stress (Figure 2b, c; Figure S1a and Table S1), suggesting it can be a promising donor for breeding to improve drought tolerance in cultivated rice varieties. Interestingly, the number of total root tips of *O. rufipogon* greatly increased after mild osmotic stress (−0.05 MPa) (Figure 2c). Stimulated root proliferation under a continuous drought treatment is a beneficial trait which can improve plant water uptake (Kerbiriou et al., 2013; Robertson et al., 1993). It has been reported in field trials that a CSSL which was derived from Nipponbare and Kasalath had a promoted branching and elongation of lateral root growth, resulting in a higher shoot dry matter than the parent Nipponbare (Kano et al., 2011). When drought becomes severe, efficient partitioning of dry matter to the shoot becomes important to maintain the yield (Kumar et al., 2006; Robertson et al., 1993). *O. rufipogon* altered allocation of carbon to the shoot growth when the stress reached −0.15 MPa, which allowed it to maintain its shoot growth nearly as well as under control conditions (Figures 2a and b). Beneficial traits inherited from *O. rufipogon* may have allowed several of the introgressed lines, notably *CUR/RUF* 47 and 11, to have superior performance to *CUR* in terms of shoot and root growth under osmotic stress (Figures 2a–c and Tables S1 and S2). *CUR/RUF* 11 has an introgression in the region 39.7–41.6 Mb on chromosome 1 (Arbelaez et al., 2015) which was identified as a QTL (39.7–40.7 Mb) correlated with number of deep roots (Courtois et al., 2009), and root dry weight, percentage of roots which are deep root, maximum root depth, and grain yield in the field conditions (Wade et al., 2015). *CUR/RUF* 47 has introgressions located at 41.6 Mb (60 bp) on the chromosome 1, 20.7–23.9 Mb on chromosome 2, and 18.6–22.1 Mb on chromosome 9 (Arbelaez et al., 2015), which cover QTLs (Chromosome 1 40–45 Mb, chromosome 2 20–25 Mb, and chromosome 9 15–20), associated with numbers of deep roots (Courtois et al., 2009). The region 18.6–22.1 Mb on chromosome 9 of *CUR/RUF* 47 also was the QTL (18.8–20.2 Mb) identified from studies on the upland rice variety Azucena as being associated with increased root length in the field (Steele et al., 2006).

*CUR/RUF* 22 was highly sensitive to osmotic stress (Figure 2a–c) with very different patterns in shoot and root growth compared to the parents *CUR* and *O. rufipogon*. The increased sensitivity may be due to interruption of genes controlling plant response to osmotic stresses. *CUR/RUF* 22 contains four introgressions (Arbelaez et al., 2015), although our data do not allow us to determine which insertion produced these new traits. Similarly, we observed a reduced shoot and root growth in *CUR/RUF* 25 and 26 under the control conditions compared to other lines (Figures 1, 2a and b). Reducing plant height has been shown to have a positive impact on rice drought tolerance (Ahmadikhah and Marufinia, 2016). In support of this idea, we found that *CUR/RUF* 26 was the only line in which shoot and root growth were not significantly affected by either osmotic stress (Figure 2a–c and Figure S1a) or ABA treatments (Figure 4a–c and Figure S1b). *CUR/RUF* 26 contains several *O. rufipogon* introgressions which do not occur in the other 6 CSSLs (Arbelaez et al., 2015). Our analysis revealed that *CUR/RUF* 26 harbors four unique introgressions on chromosome 2, one unique introgression on chromosome 5, one on chromosome 10, and two on chromosome 11. The total length of these introgressions is 13.1Mbp. Further selection to obtain single introgression CSSLs from *CUR/RUF* 26 will help to identify genomic regions controlling insensitivity of *CUR/RUF* 26 traits to ABA and osmotic treatments, potentially traits of use in the development of more drought‐tolerant cultivars of rice.

Wen and Renee (2007) observed a significant correlation of the growth of rice radicle and plumule between their responses to the ABA treatment and the osmotic stress. They also reported that these growth responses to ABA were correlated with plant yield in the field after drought stress. Although we found the shoot and root growth of *CUR/RUF* 8 showed a similar responsive pattern to the ABA treatments and osmotic stress, the shoot growth of *O. rufipogon* responded to ABA and osmotic stresses very differently (Figures 2a,b and 4a,b). It has been suggested that plant responses to osmotic stress or drought can be regulated by ABA‐dependent or by ABA‐independent pathways (Boudsocq and Laurière, 2005; Rowe et al., 2016; Tuteja, 2007; Xu et al., 2013). Therefore, it is likely that the response of shoot growth in *O. rufipogon* to osmotic stresses is regulated by an ABA‐independent mechanism. Our findings suggest that the response of rice shoot growth to ABA is genotype dependant and differ from those to osmotic, so it will be challenging to extrapolate from genotype responses to ABA to predict the impacts of abiotic stress.

It has been well documented that ABA inhibits shoot growth in general (Bensen et al., 1988; Creelman et al., 1990; Saab et al., 1990; Watts et al., 1981), although interestingly, we observed a stimulatory response of shoot growth to 0.1 µM ABA treatment in *CUR* (Figure 4a). This stimulatory effect has also been observed in wheat (Valluru et al., 2016), where it was suggested that ethylene may be involved in this response to ABA. In addition to the shoot growth, a dose‐dependent dual response was observed in the number of total root tips of *CUR/RUF* 8 (Figure 4c). The published literature indicates that the response of root branching to ABA is variable. For example, ABA inhibited lateral root development in *Arabidopsis* (De Smet et al., 2003) promoted the formation of new lateral root primordia in legume (Gonzalez et al., 2015), and increased lateral root density in rice (Chen et al., 2006; Liang and Harris, 2005) and legumes (Liang and Harris, 2005). Only one study by Gonzalez et al. (2015) reported a dose‐dependent dual effect of ABA on lateral root emergence in legume plants. Our study offers further evidence that the response of the number of total root tips to ABA can be dose dependent and nonlinear, suggesting that plant roots have plasticity to adjust their morphology in response to internal or external signals.

The CSSLs showed a range of responses to ABA signals at different concentrations; examining these responses allowed us to identify introgression lines altered in the regulation of certain traits. For example, *CUR/RUF* 47 and 19 showed very similar patterns of shoot and root growth under ABA treatments (Figures 4a and b). Based on previous published genotyping results (Arbelaez et al., 2015), both lines shared four introgressions which do not overlap with those in the other five CSSLs, two on chromosome 10 and two on chromosome 11. These four introgressions contain 169 annotated gene loci (http://rice.plantbiology.msu.edu/cgi‐bin/gbrowse/rice), although none of these encode genes that have any obvious annotated function related to ABA metabolism or signaling. The number of total root tips of CSSLs showed increases, decreases, or no response to ABA treatments (Table S2). *CUR/RUF* 22 was the only CSSL which showed the same responsive pattern of the number of total root tips as *O. rufipogon*. There are two *O. rufipogon* intogressions in *CUR/RUF* 22 which do not overlap with the other six CSSLs: one on chromosome 3 and five unique introgressions on chromosome 10. These six introgressions contain 824 annotated gene loci (http://rice.plantbiology.msu.edu/cgi‐bin/gbrowse/rice) and 245 of these have no assigned function (annotated as “hypothetical” or “expressed” protein). Further study on those segments is needed to allow us to understand the gene functions driving root branching in response to ABA in rice.

Screening of root traits has been one of the most challenging areas in phenotyping, especially when comparing across a large number of genotypes. Hydroponic systems using PEG to induce osmotic stress provide a practical solution to this challenge and have been used to identify drought tolerance–related QTLs in rice (e.g., Kato et al., 2008; Srividya et al., 2011) or drought‐tolerant genotypes (Swapna and Shylaraj, 2017). However, these systems have significant differences from the field situation, particularly in the case of rain‐fed agriculture. In the field, drought varies in both severity and duration, producing a more variable abiotic stress, and hence potentially more variable impacts on crop growth and development (Passioura, 1972). Furthermore, soil drying usually increases mechanical impedance which prevents roots from penetrating into deeper soil layers (Kato et al., 2007), a factor absent in hydroponics‐based systems. The soil environment may explain why root traits such as the thickness and angles of roots (Kato et al., 2006), root dry weight (Wade et al., 2015), and small xylem diameters in seminal roots (Polania et al., 2017) have been found to impact grain yield in the field. Despite these caveats, phenotyping root traits at early stages can be a good indicator for the performance of plants at mature stages (Kato et al., 2008), and, significantly, we demonstrate here that there are overlaps between the QTL regions identified in field‐based research as being associated with root traits which improve drought tolerance and the introgressions in our CSS lines.

## 5 CONCLUSION

Our work has highlighted the importance of evaluating varying degrees of stress to identify traits that could improve the resilience of rice to drought. We screened 48 introgressed lines, which collectively covered 97.6 % of the *O. rufipogon* genome, and identified beneficial traits in some of these lines, such as *CUR/RUF* 11 and 47, which improved their tolerance to mild osmotic stress. We also demonstrated a higher tolerance to osmotic stress in the wild ancestor *O. rufipogon* than in the modern cultivar *CUR*. Our findings suggest that the growth of modern cultivars such as IR 64, which is widely grown in Asia (Khush, 1995) but very susceptible to drought (Wade et al., 1999), could be improved by introducing chromosome segments from the wild rice, *O. rufipogon*.

## CONFLICT OF INTEREST

The authors declare no conflict of interest.

## AUTHORS' CONTRIBUTIONS

All authors made substantial contributions to the design, analysis, and interpretation of data, and were involved in drafting and discussing the manuscript for publication.

## Supporting information

Supplementary Material
