# Supplementary material for: Impact of osmotic stress on the growth and root architecture of introgression lines derived from a wild ancestor of rice and a modern cultivar
Source: Plant Environ Interact. 2020 Aug 8;1(2):122–33. doi: 10.1002/pei3.10026 (PMC10168093; doi:10.1002/pei3.10026)
Supplement: Supplementary file 1 — Supplementary Material [file PEI3-1-122-s001.docx]

Supporting information

**Figure S1**. Responses of the longest root growth to (a) three 5%, 10%, 15% PEG treatments and control treatment, (b) three 0.001 µM, 0.1 µM, and 5 µM ABA treatments and control treatment in *CUR*, *O. rufipogon* and seven CSSLs. The values are means, and the vertical bars represent standard errors. Data analysed using One-way ANOVA and different letters indicate significant differences between the PEG or ABA treatments at P <0.05. n=5 or 6.

(a)

(b)

**Table S1**. Multiple pairwise comparisons (Tukey Test) of shoot dry weight, total root length and number of the number of total root tips between genotypes *CUR*, *O. rufipogon* and *CUR/RUF* 47 under control and 3 PEG treatments. Mean differences between genotypes and *P* values are presented. Significance: *, P<0.05; **, P<0.001.

|  |  | Control (*P* value) | | | 5% PEG (*P* value) | | | 10% PEG (*P* value) | | | 15% PEG (*P* value) | | |
| --- | --- | --- | --- | --- | --- | --- | --- | --- | --- | --- | --- | --- | --- |
| Line (a) | Line  (b) | Total shoot dry weight | Total root Length | Number of total root tips | Total shoot dry weight | Total root Length | Number of total root tips | Total shoot dry weight | Total root Length | Number of total root tips | Total shoot dry weight | Total root Length | Number of total root tips |
| CUR | **Ruf 8** | 0.616 | 0.915 | 0.908 | 1 | 1 | 1 | 0.613 | 0.427 | 0.176 | 1 | 1 | 1 |
|  | **Ruf 11** | 1 | 1 | 0.998 | 0.827 | 0.979 | 1 | 1 | 1 | 0.993 | 0.144 | 0.817 | 0.923 |
|  | **Ruf 19** | 1 | 0.999 | 0.993 | 0.504 | 0.902 | 0.895 | 0.549 | 0.65 | 0.366 | 0.981 | 1 | 0.998 |
|  | **Ruf 22** | 0.95 | 1 | 0.988 | 1 | 1 | 0.191 | 0.977 | 0.992 | 0.994 | 0.999 | 0.999 | 0.999 |
|  | **Ruf 25** | 0.195 | 0.069 | 0.086 | 0.897 | 0.441 | 0.955 | 0.041 | ** | ** | 0.309 | 0.542 | * |
|  | **Ruf 26** | * | 0.148 | 0.581 | 0.011 | 0.707 | 0.999 | * | 0.001 | 0.453 | 0.064 | 0.994 | 0.741 |
|  | **Ruf 47** | 0.78 | 1 | 1 | 0.778 | 0.719 | 0.281 | 1 | 0.954 | 0.935 | * | 0.478 | 0.118 |
|  | **Ruf O** | 0.991 | 0.807 | 0.975 | ** | 0.08 | * | 0.975 | ** | ** | ** | * | ** |
| Ruf O | **Ruf 8** | 0.138 | 0.076 | 0.243 | ** | 0.118 | * | 0.067 | ** | ** | ** | 0.072 | ** |
|  | **Ruf 11** | 0.999 | 0.683 | 0.645 | * | 0.633 | * | 0.983 | ** | ** | 0.153 | 0.754 | * |
|  | **Ruf 19** | 0.855 | 0.392 | 0.549 | 0.124 | 0.837 | 0.312 | 0.051 | ** | ** | * | 0.162 | ** |
|  | **Ruf 22** | 1 | 0.985 | 1 | ** | * | 0.963 | 0.416 | ** | ** | ** | 0.184 | ** |
|  | **Ruf 25** | * | ** | * | ** | ** | ** | ** | ** | ** | ** | ** | ** |
|  | **Ruf 26** | ** | ** | 0.059 | ** | ** | * | ** | ** | ** | ** | ** | ** |
|  | **Ruf 47** | 0.998 | 0.959 | 0.998 | * | 0.959 | 0.915 | 0.994 | ** | ** | 0.66 | 0.963 | 0.145 |
| Ruf 47 | **Ruf 8** | * | 0.707 | 0.731 | 0.475 | 0.807 | 0.271 | 0.459 | * | * | * | 0.683 | * |
|  | **Ruf 11** | 0.91 | 1 | 0.975 | 1 | 0.999 | 0.62 | 1 | 0.89 | 0.453 | 0.99 | 1 | 0.809 |
|  | **Ruf 19** | 0.418 | 0.984 | 0.949 | 1 | 1 | 0.987 | 0.398 | 0.084 | * | 0.175 | 0.863 | * |
|  | **Ruf 22** | 1 | 1 | 0.999 | 0.501 | 0.334 | 1 | 0.932 | 0.493 | 0.468 | 0.076 | 0.887 | 0.424 |
|  | **Ruf 26** | ** | 0.019 | * | 0.087 | * | * | * | ** | ** | ** | * | ** |
|  | **Ruf 27** | * | 0.05 | 0.345 | ** | * | 0.695 | * | ** | * | ** | 0.072 | * |

**Table S2**. Summary of shoot and root growth response from *CUR*, *O. rufipogon* and CSSLs under 5% and 15% PEG treatments; 0.001 µM and 5 µM ABA treatments. **↓** or **↑** indicates the decrease/increase between control and the treatment is significant P<0.05; and -- indicates no significant difference.

|  | PEG treatments | | | | | | ABA treatments | | | | | |
| --- | --- | --- | --- | --- | --- | --- | --- | --- | --- | --- | --- | --- |
|  | 5% | 15% | 5% | 15% | 5% | 15% | 0.001 µM | 5 µM | 0.001 µM | 5 µM | 0.001 µM | 5 µM |
| **Line** | **Shoot dry weight** | | **Total root Length** | | **Number of total root tips** | | **Shoot dry weight** | | **Total root Length** | | **Number of total root tips** | |
| **CUR** | -- | ↓ | ↓ | ↓ | ↓ | ↓ | -- | -- | -- | ↓ | -- | -- |
| **Ruf 8** | -- | ↓ | -- | ↓ | -- | -- | -- | ↓ | -- | ↓ | **↑** | ↓ |
| **Ruf 11** | -- | ↓ | -- | ↓ | -- | -- | -- | -- | -- | -- | -- | -- |
| **Ruf 19** | -- | -- | -- | ↓ | -- | ↓ | -- | ↓ | -- | ↓ | -- | -- |
| **Ruf 22** | ↓ | ↓ | ↓ | ↓ | -- | ↓ | -- | ↓ | -- | ↓ | ↓ | ↓ |
| **Ruf 25** | -- | ↓ | -- | ↓ | -- | ↓ | -- | -- | -- | -- | -- | -- |
| **Ruf 26** | -- | -- | -- | -- | -- | -- | -- | -- | -- | -- | -- | -- |
| **Ruf 47** | -- | ↓ | -- | ↓ | -- | -- | -- | ↓ | -- | ↓ | -- | -- |
| **Ruf O** | -- | ↓ | -- | ↓ | **↑** | -- | ↓ | ↓ | -- | ↓ | ↓ | ↓ |
